# The RSSearch™ Registry: patterns of care and outcomes research on patients treated with stereotactic radiosurgery and stereotactic body radiotherapy

**DOI:** 10.1186/1748-717X-8-275

**Published:** 2013-11-25

**Authors:** Joanne N Davis, Clinton Medbery III, Sanjeev Sharma, Adnan Danish, Anand Mahadevan

**Affiliations:** 1 The Radiosurgery Society, 1350 Campbell, Suite 105, Sunnyvale, CA 95008, USA; 2 Department of Radiation Oncology, St. Anthony Hospital, Oklahoma City, OK, USA; 3 Department of Radiation Oncology, St. Mary’s Medical Center, Huntington, WV, USA; 4 Department of Radiation Oncology, Riverview Medical Center, Red Bank, NJ, USA; 5 Department of Radiation Oncology, Beth Israel Deaconness Medical Center, Harvard Medical School, Boston, MA, USA

**Keywords:** Stereotactic radiosurgery, Stereotactic body radiation therapy, Registry, Brain metastases, Lung cancer, Liver metastases, Prostate cancer, Pancreatic cancer

## Abstract

**Background:**

The RSSearch™ Registry is a multi-institutional, observational, ongoing registry established to standardize data collection from patients treated with stereotactic radiosurgery (SRS) and/or stereotactic body radiotherapy (SBRT). This report describes the design, patient demographics, lesion characteristics, and SRS/SBRT treatment patterns in RSSearch™. Illustrative patient-related outcomes are also presented for two common treatment sites – brain metastases and liver metastases.

**Materials and methods:**

Thirty-nine US centers participated in RSSearch™. Patients screened for SRS/SBRT were eligible to be enrolled. Descriptive analyses were performed to assess patient characteristics, physician treatment practices, and clinical outcomes. Kaplan-Meier analysis was used to determine overall survival (OS), local progression-free (LPFS), and distant disease-free survival (DDFS).

**Results:**

From January, 2008 – January, 2013, 11,457 patients were enrolled. The median age was 67 years (range 7–100 years); 51% male and 49% female. Forty-six percent had no prior treatment, 22% had received chemotherapy, 19% radiation therapy and 17% surgery. There were 11,820 lesions from 65 treatment locations; 54% extracranial and 46% intracranial. The most common treatment locations were brain/cranial nerve/spinal cord, lung, prostate and liver. Metastatic lesions accounted for the majority of cases (41.6%), followed by primary malignant (32.9%), benign (10.9%), recurrent (9.4%), and functional diseases (4.3%). SRS/SBRT was used with a curative intent in 39.8% and palliative care in 44.8% of cases. The median dose for all lesions was 30 Gy (range < 1 – 96.7 Gy) delivered in a median number of 3 fractions. The median dose for lesions in the brain/cranial nerve/spinal cord, lung, liver, pancreas and prostate was 24, 54, 45, 29 and 36.25 Gy, respectively. In a subset analysis of 799 patients with 952 brain metastases, median OS was 8 months. For patients with a Karnofsky performance score (KPS) > 70, OS was 11 months vs. 4 months for KPS ≤ 70. Six-month and 12-month local control was 79% and 61%, respectively for patients with KPS ≤ 70, and 85% and 74%, respectively for patients with KPS > 70. In a second subset analysis including 174 patients with 204 liver metastases, median OS was 22 months. At 1-year, LPFS and DDFS rates were 74% and 53%, respectively. LPFS.

**Conclusion:**

This study demonstrates that collective patterns of care and outcomes research for SRS/SBRT can be performed and reported from data entered by users in a common database. The RSSearch™ dataset represents SRS/SBRT practices in a real world setting, providing a useful resource for expanding knowledge of SRS/SBRT treatment patterns and outcomes and generating robust hypotheses for randomzed clinical studies.

## Introduction

Stereotactic radiosurgery (SRS) has been used to treat intracranial and spinal benign and malignant lesions, as well as functional disorders of the brain for several decades [1-4]. Lars Leksell first described SRS as a technique for the noninvasive destruction of intracranial tissue or lesions where surgery was not considered an option and designed and built the first Gamma Unit for clinical use in the 1950’s [5,6]. Between 1968–1982, the Stockholm group treated 762 patients with intracranial lesions including meningiomas, pituitary tumors, pineal gland tumors, acoustic tumors, arterio-venous malformations (AVM), and functional diseases including trigeminal neuralgia, anxiety and compulsive disorders [6]. The technology continued to evolve with the development of linear accelerator-based radiosurgery [7], frameless radiosurgery, three dimensional computerized treatment planning systems and image guidance, which led to wide-spread use of SRS for the treatment of intracranial lesions. SRS is now used as an alternative to surgical resection for poorly accessible intracranial lesions and in eloquent areas, primary therapy for benign and recurrent tumors and adjuvant treatment for post-surgical residual lesions. The SRS dose typically ranges from 12–24 Gy for benign and malignant lesions and is dependent on lesion location, size and distance to critical normal structures. Recent studies have shown SRS can achieve similar local control rates to surgical resection for meningiomas and > 90% 5-year local control rates for pituitary adenomas, acoustic neuromas [8-10].

In the early 1990’s, the concept of stereotactic body radiotherapy (SBRT), delivering a high dose of radiation to an extracranial target within the body in either a single or a few fractions was developed from the techniques and procedures of SRS [11]. Initial studies further developed the techniques to deliver SBRT to lung and liver lesions [12-14]. SBRT is characterized by patient immobilization, target localization and tracking software, limiting high doses of radiation to normal tissues, accounting for organ motion and sub-millimeter accuracy. Improvements in all these areas with rapid advances in SRS/SBRT technology have resulted in expansion of SBRT clinical applications and an exponential increase in patients treated. In the past decade, SBRT has been used as a treatment for lesions of the prostate [15-19], pancreas [20-23], head and neck [24-26], kidney [27], breast [28], and gynecological tumors [29-31].

Despite increased knowledge of the technology and clinical outcomes reported from single institutions, information on physician practice patterns of SRS and SBRT in the daily clinical practice is limited. A registry provides a systematic and inclusive database of information which can reveal and evaluate the effectiveness of management practices in the real world [32]. In contrast to a clinical trial, where patient enrollment is defined by specific inclusion and exclusion criteria, treatment is dictated by protocol guidelines, and treatment evaluation is measured at specific follow-up time intervals, a registry documents actual care, representing a broad spectrum of patients where treatments are not specified by protocol guidelines and patient follow-up schedules are conducted in a real-life setting. A registry can provide information as to whether clinicians are adhering to practice guidelines, may complement randomized clinical trials and/or identify new clinical applications and treatment benefits [33-35]. Such patterns of care, utilization and outcomes can help design and conduct future trials more effectively.

The RSSearch™ Registry is an ongoing, observational, multi-institutional registry collecting patient and tumor characteristics, treatment plan and treatment delivery information, toxicity, and outcome data from patients treated with SRS/SBRT. This report describes the design and methods of the registry, the baseline demographics and clinical characteristics of the enrolled patients and SRS/SBRT treatment management patterns. It also illustrates the feasibility of generating useful outcomes data outside the confines of tightly controlled clinical trials.

## Materials and methods

The RSSearch™ Registry was conceptualized and designed by a board of RSSearch™ Clinical Advisors in 2006. The RSSearch™ Clinical Advisory Committee is comprised of radiation oncologists, neurosurgeons, surgeons, medical oncologists, and medical physicists to create and oversee the scientific conduct of the registry. The goals and objectives are to provide a method to collect standardized data on the use of SRS/SBRT treatment practices and outcomes to help determine the most effective clinical use of SRS/SBRT in management of patients with life threatening tumors and other diseases. Data collected in RSSearch™ includes patient demographics, tumor/lesion characteristics, treatment locations, treatment plan and treatment delivery information, toxicity, and clinical outcomes, including symptom control, lesion response, patient survival, and disease progression.

Through an initial grant provided by Accuray Incorporated (Sunnyvale, CA), a third-party medical software and web management company, Advertek Inc. (Louisville, KY), was contracted to provide services to design, store, and maintain the web-based database. The RSSearch™ Registry is currently managed by the Radiosurgery Society®, a multi-disciplinary non-profit organization aimed at advancing the science and clinical practice of radiosurgery [36]. Additional clinical oversight is provided by the RSSearch™ Clinical Advisory Committee. The database meets all requirements to comply with the Health Insurance Portability and Accountability Act to maintain system security, transmission of data and patient confidentiality. Select patient identifying information is recorded in RSSearch™ as needed for registry operations, but is visible to the participating site only and further protected by robust security systems put in place by Advertek. A role-based security model is implemented to restrict access to only the appropriate information based on the user’s role in the registry. Quality assurance measures have been built into the system to reduce error and redundancy. These include explicit definitions for each question and set of variables to help reduce interpretive error and improve quality control. Electronic data ranges, logic checks and data entry requirements are implemented within the system to reduce entry of incorrect or duplicate data. All participants are trained on the system.

All centers treating patients with SRS/SBRT clinically are offered and encouraged to participate. Participation is voluntary and no compensation is provided either to patients or participating centers. Each principal investigator is provided a copy of the RSSearch™ Registry protocol, case report forms, sample patient informed consent, and web-based training for data entry and database navigation. Institutional Review Board (IRB) approval is required at all participating centers. All patients who are screened for potential SRS/SBRT treatment are eligible to be included in the RSSearch™ Registry. All prospective patients are required to sign an informed consent, as required by individual IRBs, prior to the patient’s data entered into the RSSearch™ Registry. Retrospective analysis of RSSearch™ is conducted from prospectively entered data. Data is entered into RSSearch™ voluntarily per institutional guidelines. Individual sites have access to their own individual dataset. The RSSearch™ administrator has access to de-identified aggregate data for quality assurance purposes and data are reviewed periodically for data completeness. Requests for de-identified aggregate data can be submitted by RSSearch™ participants to the RSS and requests are reviewed by the RSSearch™ Review Committee.

Patient demographics are captured during the screening process and include gender, ethnicity, age, weight, height, smoking history and Karnofsky performance score. Information on referral sources, primary and secondary payer information, previous treatments, and co-morbidities are also captured during the screening process. SRS/SBRT treatment sites are classified using the World Health Organization (WHO) International Classification of Diseases (ICD), version 9 codes. Tumor characteristics including TNM stage, histology/cytology, lesion size, tumor markers and data from diagnostic imaging are recorded. All patients were treated with the CyberKnife™ Robotic Radiosurgery System, Accuray Inc., Sunnyvale, CA. Both manual and automatic treatment planning upload capabilities are available in RSSearch™ to capture treatment planning and delivery information. Treatment planning data fields include treatment planning system version, method of dose calculation, dose optimization method, number of fractions, number of fiducials, path set, tracking method, number of monitor units, prescription dose, maximum dose, number of nodes, collimator type and size, doses to organs at risk, treatment times, set-up times, and delivery times and are captured for each treated lesion.

RSSearch™ has an extensive outcome and follow-up data section that captures toxicity, lesion response, disease-progression, tumor markers, surrogate endpoints, survival, information from post-treatment imaging, and additional treatments. Toxicity reporting utilizes the Common Toxicity Criteria for Adverse Event Reporting, version 3. Patient demographics, lesion characteristics, and treatment management practices were examined using descriptive statistics using GraphPad and Instat Software, La Jolla, CA. Overall survival was calculated from the date the patient was evaluated for SBRT. Local failure and distant failure patterns were determined from the date of SBRT evaluation to the first date of physician reported-failure. Distant disease included disease outside of the local treatment area. Survival curves were plotted based on the Kaplan-Meier method.

## Results

### Patient characteristics

Between January 2008 and January 2013, 11,457 patients from 39 participating centers in the US were enrolled in the RSSearch™ Registry. Ninety-two percent of the subjects were enrolled prospectively and 7.8% of the subjects had their data retrospectively entered into the database. The median age of the patients at time of enrollment was 67 years (range 7–100); with 89% of patients age 50 years and older (Table 1). Fifty-one percent of the patients were male and 49% were female. Eighty-nine percent of the patients were Caucasian, 6.6% African-American, 1.8% Hispanic, and 0.8% Asian. The majority of patients had excellent baseline performance status with a median Karnofsky score of 90% (10–100).

**Table 1:** Patient characteristics and demographics

**Variable (N)**	**N (%)**
Patients enrolled	11457
Gender (11345)	
Male	5836 (51%)
Female	5509 (49%)
Median age (range), years	67 (7 – 100)
Age groups (10886)	
< 20	21 (0.2%)
20 – 29	115 (1.1%)
30 – 39	235 (2.2%)
40 – 49	829 (7.6%)
50 – 59	1879 (17.3%)
60 – 69	3083 (28.3%)
70 – 79	3183 (29.2%)
≥ 80	1541 (14.2%)
Median weight (pounds)	170
Median height (inches)	67
Median Karnofsky score (range)	90 (10–100)
Race/ethnicity (10352)	
Caucasian	9269 (89.5%)
Black/African American	682 (6.6%)
Hispanic	185 (1.8%)
Asian	83 (0.8%)
Pacific-Asian Islander	13 (0.1%)
Other	51 (0.5%)
Don’t know	69 (0.7%)
Primary health insurance (10547)	
Private	40%
Medicare	54.4%
Self-pay/none	0.7%
Patients not treated with SRS/SBRT	1060 (9%)
Reason for not completing SRS/SBRT (1060)	
SRS/SBRT determined not clinically appropriate after further review	272 (25.7%)
Patient has extensive disease	226 (21.3%)
Patient declined SRS/SBRT	148 (14.0%)
Watchful waiting indicated	109 (10.3%)
Payment not pre-authorized	53 (5.0%)
Alternative insurance-covered treatment elected	44 (4.2%)
Other	29 (2.7%)
Patient not able to tolerate SRS/SBRT	16 (1.5%)
Co-morbidities rule out SRS/SBRT	15 (1.4%)

The majority of patients were referred to SRS/SBRT treatment centers from medical oncologists (30%), neurosurgeons (17%), radiation oncologists (11%), urologists (8%), and cardio-thoracic surgeons (5%). Other referral specialties included pulmonology (4%), primary care (2%), neurology (1%), general surgery (1%), and gynecology (0.5%). Four percent of the patients were self-referred. Medicare was listed as the primary health insurance payer for 54.4% of the patients, private insurance for 40.8% of the patients, and 0.7% of the patients were uninsured or paid out-of-pocket (Table 1). At the time of the analysis, 1060 (9%) of the screened patients did not undergo SRS/SBRT treatment. Reasons for not completing SRS/SBRT treatment included those deemed inappropriate after further review (272 patients; 25.7% of non-treated patients), extensive disease (226 patients; 21.3% of non-treated patients), or patient decision not to pursue treatment (148 patients; 14.0% of non-treated patients); additional reasons are listed in Table 1.

Forty-six percent of patients registered had no prior treatment, 22% had previous chemotherapy, 19.1% had received prior external beam radiation therapy and 17.7% had undergone surgery (Table 2). Nearly fifteen percent of patients were considered surgically inoperable at the time of enrollment and 10% of cases were considered medically inoperable. The primary baseline co-morbidities reported for medically inoperable patients included pulmonary (64.9%), cardiac (17.3%), vascular (3.5%) and neurological (1.9%). Age was also reported as a contra-indication for 2% of medically inoperable patients.

**Table 2:** Clinical characteristics of the patients enrolled at baseline

**Variable (N)**	**N (%)**
Number of screened entries	11820
Prior Treatment(s)	
None	5399 (45.7%)
Chemotherapy	2597 (22.0%)
External beam radiation	2262 (19.1%)
IMRT	239 (2%)
CyberKnife Robotic Radiosurgery	419 (3.5%)
Other radiosurgery	120 (1.0%)
Proton therapy	7 (0.1%)
Brachytherapy	34 (0.3%)
Surgery	2096 (17.7%)
Hormone therapy	179 (1.5%)
Cryotherapy	11 (0.1%)
Immunotherapy	7 (0.1%)
Other treatment	443 (3.8%)
Surgically inoperable	1728 (14.6%)
Medically inoperable	1188 (10.1%)
Co-morbidities listed for medically inoperable patients (N = 1188)*	
Age	24 (2%)
Cardiac	206 (17.3%)
Neurological	22 (1.9%)
Pulmonary	771 (64.9%)
Vascular	41 (3.45%)

* Co-morbid conditions are not mutually exclusive.

### Tumor characteristics

**Figure 1:**
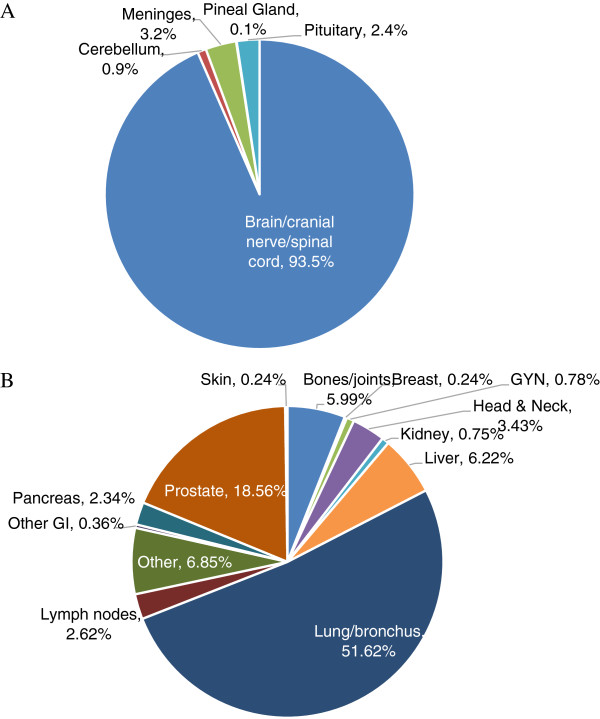
Location and percentage of intracranial lesions (A) and extracranial lesions (B) treated with SRS/SBRT.

At the time of this analysis, data were available on 11,820 lesions. A description of the lesion characteristics are shown in Table 3. The most prevalent lesion type was metastatic lesions (41.6%), followed by primary malignant lesions (32.9%) benign lesions (10.9%), recurrent primary lesions (9.4%), functional diseases (4.3%) and arterio-venous malformations (0.8%). In total, there were 65 different SRS/SBRT anatomical treatment sites. The majority of lesions were extracranial (54% extracranial vs. 46% intracranial), but the predominant single treatment location category was brain/cranial nerve/spinal cord which represented 42.3% of all cases and 93% of intracranial lesions. The distribution of intracranial lesions by location is shown in Figure 1. Three percent of all intracranial cases were located in the meninges, 2.4% in the pituitary, 0.9% in the cerebellum and 0.1% in the pineal gland. The distribution of intracranial lesion by pathology/histology is shown in Table 3. Metastases were the most prevalent type of intracranial lesion (53.6%) with adenocarcinoma and squamous cell carcinoma as the most common histology. The second most prevalent intracranial type of lesion was benign (21.6%) and the most common benign histology was non-malignant meningioma (360 cases, 6.7% of intracranial lesions) and acoustic neuroma (321 cases, 5.9% of intracranial lesions). Recurrent tumors account for 4.8% of intracranial lesions with gliobastoma as the most prevalent recurrent tumor followed by meningioma, astrocytoma and pituitary adenoma. Primary malignant tumors were the least prevalent type of intracranial lesion and account for 4.2% of intracranial cases. One half of all primary malignant intracranial lesions fell into the broad category glioma, with glioblastoma and astrocytoma as the most prevalent types (Table 3). For the age group of 0–19 years, 18 of 21 lesions were located in brain/cranial nerve/spinal cord and the most prevalent histology for this age group was arterio-venous malformations, glioblastoma and astrocytoma.

**Table 3:** Lesion characteristics and most common lesion location and histology

**Variable (N)**	**N (%)**
**All lesions – lesion type (11154)**	
Arterio-venous malformation	92 (0.8%)
Benign tumor	1218 (10.9%)
Malignant primary tumor	3668 (32.9%)
Metastatic tumor	4639 (41.6%)
Recurrent primary tumor	1050 (9.4%)
Functional disease	485 (4.3%)
**Intracranial lesions (5441)**	
Benign lesions	1176 (21.6%)
Acoustic neuroma	321 (5.9%)
Meningioma	360 (6.7%)
Benign, NOS	155 (2.9%)
Pituitary adenoma	88 (1.6%)
Primary malignant	226 (4.2%)
Astrocytoma	29 (0.6%)
Glioblastoma	76 (1.4%)
Glioma	8 (0.2%)
Meningioma, malignant	44 (0.8%)
Metastatic	2917 (53.6%)
Brain/cranial nerve/spinal cord	2867 (52.7%)
Meninges	4 (0.8%)
Recurrent	263 (4.8%)
Astrocytoma	19 (0.4%)
Glioblastoma	87 (1.6%)
Glioma	8 (0.2%)
Pituitary adenoma	16 (0.3%)
Functional disease	485 (9%)
Trigeminal neuralgia – typical	364 (6.7%)
Trigeminal neuralgia – atypical	99 (1.8%)
Trigeminal neuralgia- MS	13 (0.3%)
**Extracranial lesions (6379)**	
Benign	42 (0.7%)
Head & neck	17 (0.3%)
Bones/joints	9 (0.2%)
Other nervous system	6 (0.1%)
Primary malignant	3442 (53.9%)
Lung/bronchus	1973 (30.9%)
Prostate	1165 (18.3%)
Pancreas	108 (1.7%)
Liver	50 (0.8%)
Metastatic	1722 (27%)
Lung/bronchus	601 (9.4%)
Bones/joints	338 (5.3%)
Liver	331 (5.2%)
Lymph node	150 (2.4%)
Recurrent	787 (12.4%)
Lung/bronchus	520 (8.2%)
Head and neck	98 (1.5%)
Gynecological	25 (0.4%)
Pancreas	21 (0.3%)

Trigeminal neuralgia was the most prevalent type of functional disease (476 cases and 98% of functional diseases). Typical and atypical trigeminal neuralgia account for 75% and 20% of functional diseases, respectively (Table 3) and trigeminal neuralgia associated with multiple sclerosis was reported in 13 cases.

The distribution of extracranial lesions by site is shown in Figure 1B. Lung/bronchus was the most prevalent extracranial location (51.6% of extracranial cases), followed by prostate (18.6%), liver (6.2%), bones/joints (6.0%), head and neck (3.4%) lymph nodes (2.6%), and pancreas (2.3%). Fifty-four percent of extracranial lesions were primary malignant, 27% metastatic, 12.4% recurrent and 0.7% benign (Table 3). There are 73 different histological classifications for primary lesions. The most common histology was adenocarcinoma (46.9%) followed by non-small cell carcinoma (16.7%) and squamous cell carcinoma (15.0%). Other histologies included hepatocellular carcinoma, small cell carcinoma, renal cell carcinoma, and cholangiocarcinoma. The most common primary site of the metastatic lesion was from lung/bronchus (19%), followed by large intestine (15%), breast (11%), gynecological tumors (10%), head and neck (9%), rectum (6%) and kidney (5%). Lung/bronchus and head and neck lesions accounted for the most prevalent recurrent cases.

### Treatment characteristics

SRS/SBRT was the primary treatment for 70% of lesions, adjuvant treatment for 14.3%, and used as a boost treatment for 9.7% of lesions (Table 4). The primary treatment objective of SRS/SBRT treatment was palliative for 44.8% and curative for 39.8% of lesions. SRS/SBRT treatment plan information was available for 8589 lesions. The radiation dose delivered to the target varied significantly across treatment locations, with the overall median dose of 30 Gy (range < 1 – 96.7 Gy) and the median number of fractions delivered was 3.

**Table 4:** SRS and SBRT treatment indication and characteristics

**Variable (N)**	**N (%)**
SRS/SBRT treatment indication (11072)	
Primary treatment	7748 (70.0%)
Adjuvant treatment	1585 (14.3%)
Non-surgical boost	1077 (9.7%)
Post-operative treatment	231 (2.1%)
Post-operative for residual tumor	336 (3.0%)
Post-operative for cavity boost	83 (0.7%)
Pre-operative treatment	12 (0.1%)
SRS/SBRT treatment objective (11072)	
Curative	4407 (39.8%)
Palliative	4958 (44.8%)
Other	564 (5.1%)
Not reported	1461 (12%)
Mean lesion volume, range (5890)	33.67 (0–5255 cc)
Median lesion size in x,y,z, mm	27.2, 24.6, 24.9
Median number of fiducials (range)	1 (1–9)
Median number of fractions (range)	3 (0–46)
Median prescription dose, range (8585)	30 (0 – 96.7 Gy)
Median maximum point dose, range (7953)	40.98 (0–100 Gy)
Most common collimator size, intracranial lesion	10 mm
Most common collimator size, extracranial lesion	20 mm

For this analysis, we identified five common treatment locations (brain, lung, liver, pancreas and prostate) to report the treatment volume, prescription dose, number of fractions and the maximum point dose to the organs at risk (Table 5). For brain/cranial nerve/spine lesions, the median volume was 3.3 cc (range < 1 – 804 cc), the median prescription dose was 24 Gy (range 2 – 96 Gy) and median number of fractions was 1 (range 1 – 25). The median maximum dose was 5 Gy to the brainstem, 0.4 Gy to the lens, 2 Gy to the optic chiasm and 1 Gy to the optic nerve. For lung lesions, the median volume was 14 cc (range 0.2 – 1751 cc), the median prescription dose was 54 Gy (range 11 – 80 Gy), and the median number of fractions was 3(range 1 – 10). The median maximum dose was 11 Gy to the esophagus, 14 Gy to the heart, 12 Gy to the trachea/bronchus, and 7 Gy to the spinal cord. For liver lesions, the median volume is 32 cc (range 1 – 877 cc), the median prescription dose is 45 Gy (range 10 – 60 Gy) and median number of fractions is 3 (range 1 – 5). The median maximum dose was 60 Gy for uninvolved liver, 17 Gy to the bowel, 6 Gy to the kidney, and 4 Gy to the spinal cord. For lesions in the pancreas, the median volume is 34 cc (range 2 – 172 cc), the median maximum dose was 29 Gy (range 2 – 60 Gy) and the median number of fractions is 3 (range 1–5). The median maximum dose was 24 Gy to the bowel, 5 Gy to the kidney, 22 Gy to the liver and 4 Gy to the spinal cord. For the prostate, the median volume was 56 cc (range 6 – 296 cc), the median dose was 36.35 Gy (6.5 – 80 Gy) delivered in 5 (range 1 – 38) fractions. The median maximum dose was 41 Gy to the bladder, 14 Gy to the femoral heads, 27 Gy to the penile bulb, 38 Gy to the rectum and 43 Gy to the urethra.

**Table 5:** Doses for common treatment sites and organs at risk reported in RSSearch™

**Treated organ**	**Organs at risk**	**Median volume (range), cc**	**Median # fractions (range)**	**Median dose (range), Gy**	**Median max point dose (range), Gy**
**Brain**		3.3 (< 1 – 804)	1 (1 – 25)	24 (2 – 96)	30 (0 – 96)
	Brain stem				5 (0 – 70)
	Eye/lens				0.4 (0 – 70)
	Optic chiasm				2 (0 – 51)
	Optic nerve				1 (0 – 41)
**Lung/bronchus**		14 (0.2 – 1751)	3 (1 – 10)	54 (11 – 80)	71 (0 – 100)
	Esophagus				11 (0 – 63)
	Heart				14 (0 – 64)
	Trachea/bronchus				12 (0 – 76)
	Spinal cord				7 (< 1– 76)
**Liver**		32 (1– 877)	3 (1– 5)	45 (10 – 60)	58 (12 – 94)
	Uninvolved liver				60 (18 – 75)
	Bowel				17 (0 – 66)
	Kidney				6 (0 – 64)
	Spinal cord				4 (0 – 17)
**Pancreas**		34 (2 – 172)	3 (1 – 5)	29 (2 – 60)	38 (0 – 73)
	Bowel				24 (0 – 56)
	Kidney				5 (0 – 27)
	Liver				22 (2 – 70)
	Spinal cord				4 (0 – 13)
**Prostate**		56 (6 – 296)	5 (1– 38)	36.25 (6.5 – 80)	48 (9 – 86)
	Bladder				41 (0.5 – 80)
	Femoral heads				14 (0.5 – 111)
	Penile bulb				27 (0.7 – 66)
	Rectum				38 (6 – 78)
	Urethra				43 (8 – 83)

### Outcomes assessment

**Figure 2:**
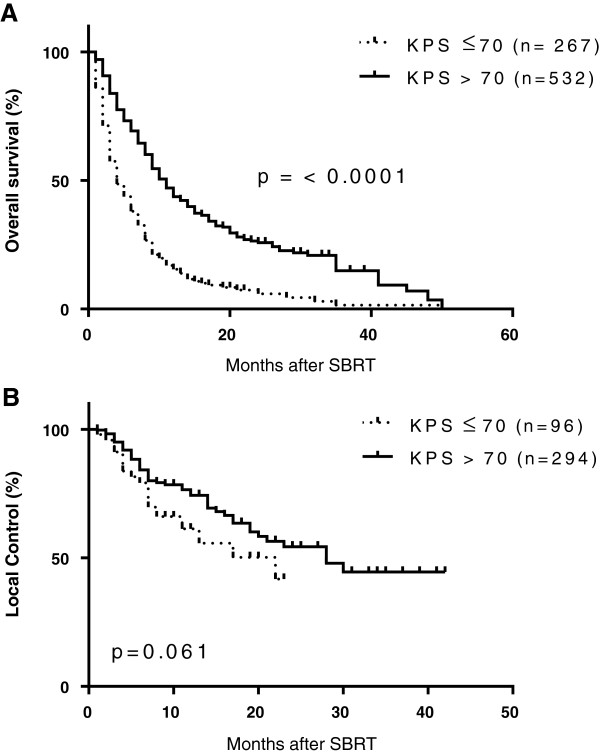
**Kaplan-Meier analysis of overall survival (A) and local control (B) of brain metastases treated with SRS.** Patients with a baseline Karnofsky performance score (KPS) ≤ 70 are indicated by dotted line and patients with KPS > 70 are indicated by solid black line.

The goal of systematic collection of data in registries is to track procedure and patient-related outcomes in order to identify areas of success and opportunities for improvement and utilization of resources. Outcomes for overall survival and disease control are shown for one common intracranial treatment site, brain metastases and one common extracranial treatment site, liver metastases. Patient demographics and treatment characteristics for 799 patients with 952 brain metastases are shown in Table 6. The number of lesions ranged from 1–7 and median tumor size was 1.2 cm (range 0.1-34 cm). The majority of metastases were from primary lung cancer (53.8%) and breast cancer (15.1%), followed by melanoma (9%) and renal (5%) tumors. Patients were treated with a median SRS dose of 22 Gy (range 2.4 – 45 Gy) delivered in a median of 1 fraction (range 1 – 4). The median overall survival rate for this cohort was 8 months. When stratified by baseline performance score, patients with good performance status with KPS > 70 had a median overall survival rate of 11 months compared to 4 months for patients with KPS ≤ 70 (p < 0.0001 by log-rank test), as shown in Figure 2. The 3-month, 6-month and 12-month local control rates for patients with KPS ≤ 70 were 91%, 79% and 61% respectively. For patients with KPS > 70, the 3-month, 6-month and 12-month local control rates were 95%, 85% and 74%, respectively. These results are comparable to rates reported from single institution studies and cooperative group studies such as RTOG 95–08, that evaluated SRS for the treatment of brain metastases [37-40].

**Table 6:** Patient and treatment details of SRS for brain metastases

**Variable (N)**	**N (%)**
Gender (799 patients)	
Male	354 (44%)
Female	445 (56%)
Median age (range), years	63 (23 – 94)
Median Karnofsky score (range)	80 (30–100)
Previous treatment	
Surgery	167 (18%)
External beam radiation	361 (37%)
Radiosurgery	111 (12%)
Chemotherapy	276 (29%)
Other	12 (1%)
None	338 (36%)
Primary tumor site (952 lesions)	
Lung	512 (53.8%)
Breast	144 (15.1%)
Melanoma	88 (9.2%)
Renal	48 (5.0%)
Colorectal	40 (4.2%)
Head and neck	30 (3.2%)
Gynecological	15 (2.0%)
Other	75 (7.8%)
Median lesion volume, cc	2.1 (0.1 – 113)
Median lesion size, cm	1.2 (0.1 – 34)
Median number of lesions (range)	1 (1 – 7)
Median number of fractions	1 (1 – 5)
Median dose (range), Gy	22 (2..4 – 45)
Prescription dose range (n = 924)	
≤ 18 Gy	223 (24%)
19-20 Gy	174 (19%)
21-22 Gy	187 (20%)
22.5 – 24 Gy	189 (20%)
25 – 30 Gy	132 (14%)
> 30 Gy	18 (2%)

**Figure 3:**
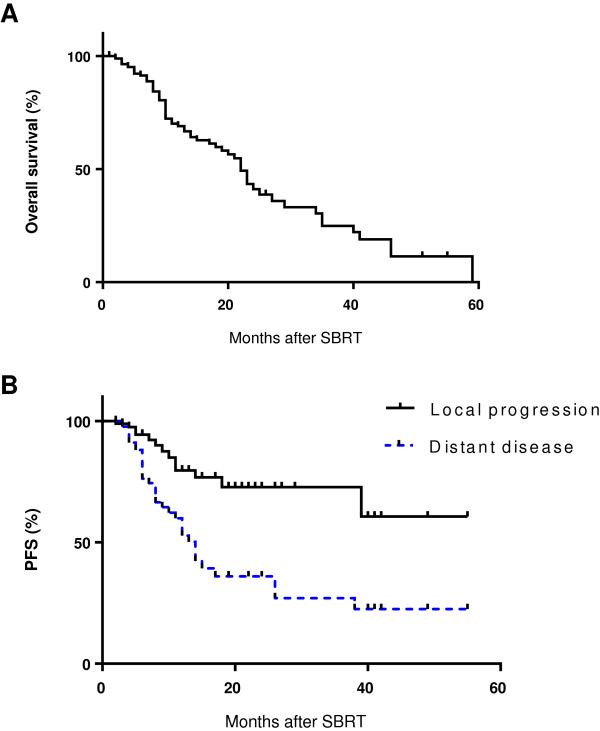
Kaplan-Meier analysis of overall survival (A) and local and distant progression-free survival (B) of hepatic metastases treated with SBRT.

Patient demographic and treatment characteristics for 174 patients with 204 liver metastases are shown in Table 7. The median follow-up was 11 months (range 1–59 months). The median lesion size was 2.7 cm (range 0.12 – 12.3 cm) and median lesion volume was 27.3 cc (range 1 – 606 cc). Fifty percent of the patients had liver metastases from colorectal cancer, 10.8% from lung, and 8.8% from breast cancer. Patients were treated with SBRT doses of 10 – 61 Gy (median 45 Gy) delivered in 1–5 fractions (median 3 fractions). Kaplan-Meier survival curves are shown in Figure 3. Median overall survival was 22 months and overall survival rates at 6, 12 and 18 months were 91%, 69% and 60%, respectively. Local progression-free survival at 6, 12 and 18 months was 94%, 76% and 70%, respectively. Distant disease progression-free survival at 6, 12 and 18 months was 77%, 53% and 37%, respectively. The median time to distant progression was 14 months. These results demonstrated that patient-related outcomes can be performed from RSSearch™ Registry data. Future outcome measures could be evaluated for all treatment sites including surrogate outcome measures like biochemical progression free survival (prostate cancer) and neurological function preservation (acoustic neuroma).

**Table 7:** Patient and treatment details of SBRT for liver metastasis

**Variable (N)**	**N (%)**
Gender (174 patients)	
Male	95 (54%)
Female	79 (45%)
Median age (range), years	69 (41 – 91)
Median Karnofsky score (range)	90 (50–100)
Previous treatment	
Surgery	22 (12%)
External beam radiation	7 (4.0%)
Radiosurgery	8 (4.6%)
Chemo embolization	1 (0.6%)
Radiofrequency ablation	5 (2.9%)
Chemotherapy	103 (59.2%)
None	54 (31.0%)
Primary tumor site (204 lesions)	
Colorectal	103 (50.5%)
Lung	22 (10.8%)
Breast	18 (8.8%)
Pancreas	9 (4.4%)
Gynecological	9 (4.4%)
Gastric	9 (4.4%)
Head and neck	7 (3.4%)
Malignant melanoma	4 (2.0%)
Anal	3 (1.5%)
Kidney	2 (1%)
Intrahepatic bile duct	2 (1%)
Other	14 (6.9%)
Median lesion volume, cc	27.3 (1–606)
Median lesion size, cm	2.7 (0.12 – 12.3)
Median number of lesions (range)	1(1–4)
Median number of fractions	3 (1–5)
Median dose (range), Gy	45 (10 – 61)
Prescription dose range	
10 – 30 Gy	35 (16.7%)
33 – 39 Gy	45 (21.6%)
40 – 45 Gy	42 (20.6%)
46.5 – 48 Gy	18 (8.8%)
51 – 54 Gy	38 (18.6%)
57 – 60 Gy	28 (13.7%)

## Discussion

The Agency for Healthcare Research and Quality (AHRQ) defines a patient registry as “an organized system that uses observational study methods to collect uniform data (clinical and other) to evaluate specified outcomes for a population defined by a particular disease, condition, or exposure, and that serves one or more predetermined scientific, clinical, or policy purposes” [32]. Registry use occurs on a variety of levels, ranging from clinic logs to rare disease entities to international disease databases. Data collected often include demographic information such as sex and age, medical history, diagnostic information, procedure and device specifics, and clinical outcomes. The uniform collection of these data creates a better understanding of practice characteristics, treatments, diseases, and outcomes. In their most practical use, registries can help identify which patients have a certain condition or disease, or follow patients receiving certain devices or treatments. The RSSearch™ Registry fulfils this ideal for patients treated with SRS and SBRT.

Multi-institutional, observational patient registries can be powerful tools to describe disease presentation, patterns of care, treatment effectiveness, safety and quality of care in a real-world setting [33-35]. The Society of Thoracic Surgery Cardiac Surgery National Database, which began in 1989 and includes over five million patient records, has been used to report treatment patterns, support quality improvement initiatives, develop clinical treatment guidelines, support comparative effectiveness studies, government collaborations, regulatory compliance and reimbursement strategies for cardiac and thoracic patients [34,41-43]. The American Society of Breast Surgeons (ASBS) MammoSite™ Registry began in 2002, as a manufacturer-sponsored registry developed to provide information on the optimal use of the device in clinical practice [35]. The ASBS assumed responsibility of the database in 2003, and have reported outcomes from the registry demonstrating efficacy, safety and improved quality of life for early-stage breast cancer patients treated with breast-conserving therapy.[44,45] The ASBS have been consistently following-up and reporting on the use of MammoSite™ as the registry matures and continues to report on clinical outcomes of partial breast irradiation even before formal prospective data from randomized clinical trials becomes available [46,47]. Clinical outcome data from registries have also been used to determine coverage (payment) policies. The Centers for Medicare & Medicaid Services expanded its coverage for PET scans in diagnosis of certain cancers because of information obtained through the National Oncologic PET Registry [33]. These reports demonstrate the potential impact registries can provide to the medical community, patient care and treatment practices.

In this report, we demonstrate that patterns of care for SRS/SBRT can be collected in a multi-institutional database and patterns of care research can be performed. We describe the initial patient demographics, lesion characteristics and SRS/SBRT treatment for 11,457 patients enrolled in the RSSearch™ Registry. To our knowledge, this is the largest aggregate report of SRS/SBRT-treated patients in a published registry. As one might expect, lesions in the brain/cranial nerve/spinal cord were the single most prevalent treatment location in RSSearch™, which correlates with the long history of SRS experience for intracranial lesions. The median SRS dose and fractionation schedule for brain lesions reported in RSSearch™ was 24 Gy delivered in 1 fraction. A single fraction of 24 Gy is the recommended dose used in to treat 1–3 metastatic brain lesions up to 2 cm as described in RTOG 9508 and RTOG 9005 [37,48] and SRS delivered as a single fraction (15–24 Gy) to individual intracranial lesions has been established as a safe alternative to surgical resection [39,49]. In a subset analysis, we reported a median overall survival rate of 11 months for patients with brain metastases with a good performance score (KPS > 70) compared to 4 months for patients with KPS ≤ 70. Outcomes for patients with brain metastases are generally poor with a median survival following whole brain radiation therapy (WBRT) alone of 3–6 months [37,39,50]. The addition of SRS following WBRT and SRS alone have improved overall survival and local control rates. Rades et al. showed a 13 month overall survival rate for SRS alone compared to 7 months for WBRT [40]. One-year local control rate for SRS alone was 64% compared to 26% for WBRT. Li et al. compared WBRT vs. SRS vs. WBRT plus SRS and showed a median survival rate of 5.7 months, 9.3 months and 10.6 months, respectively [51]. KPS has been shown to be a predictor of survival for patients with brain metastases [52]. Using recursive partitioning analysis of three RTOG trials, the best survival (median 7.1 months) was observed in patients < 65 years, KPS of at least 70 and a controlled primary tumor with brain as the only site of metastases. The worst survival (median 2.3 months) was seen in patients with KPS < 70. In our study, overall survival was significantly greater in patients with KPS > 70 compared to KPS ≤ 70, correlating with previous reports.

When using high doses of radiation with SRS/SBRT, there is critical importance to minimize the volume of normal tissues receiving high dose per fraction in order to protect normal tissues from adverse radiation effects. The American Association of Physicists in Medicine (AAPM) task group and others have defined normal dose limits for organs at risk for SBRT from published literature and randomized clinical trials [53-55]. We calculated the median maximum point dose for organs at risk from the treatment plans in RSSearch™ in order to compare to reported treatment guidelines. For the brainstem, eye/lens, optic chiasm and optic nerve, the median max point dose in RSSearch™ was 5, 0.4, 2 and 1 Gy, respectively, which is below the recommended max point dose ranges of 15–31 Gy for the brainstem, 2–3 Gy for the eye/lens, 10–15 Gy for optic chiasm and 8–15 Gy for optic nerve when delivered in 1 fraction [53,54]. Furthermore, with automated data entry from treatment platforms, more stringent dose-volume histogram analysis of all targets and organs at risk can be performed. These results indicate that most participating centers treating intracranial lesions adhere to recommended guidelines for tolerance limits for normal organs.

In this analysis, we also examine patient demographics, treatment practices and outcomes of liver metastases, a common extracranial treatment location. Currently, there is no consensus for the treatment of primary liver tumors and liver metastases with radiation. In 2012, the Liver Cancer Workgroup of the Third International Consensus on Metastases Workshop at the 2010 American Society for Radiation Oncology meeting published an international survey on the status of radiation therapy of liver metastases [56]. The survey indicated there was a 54% increase in the average number of liver referrals over the past 5 years and the majority of referrals were for SBRT. No uniform SBRT treatment dose was identified and there was a wide variation of treatment regimens which were dependent on whether the treatment objective was curative or palliation. The group concluded there is a need for prospective studies and registries for comparison of treatment regimens and identification of parameters to optimize patient selection. Interestingly, liver is the third most common extracranial SBRT treatment location in RSSearch™ and SBRT is being used as both a palliative and curative treatment option for liver lesions. The SBRT dose ranged from 10 – 60 Gy delivered over 1–5 fractions, with the median SBRT dose of 45 Gy delivered in 3 fractions. The median OS was 22 months and OS 6, 12 and 18 months were 91%, 69% and 60%, respectively. LPFS at 6, 12 and 18 months was 94%, 76% and 70%, respectively. In our analysis of this patient group (n = 174) with a wide variety of underlying primary tumors, different systemic and local treatments, lesion sizes and dose/fractionation schedules the OS and local control rates were within the range of published reports[57,58]. Future studies are planned to examine prognostic factors and the effect of dose/fractionation schedules on OS and local control from the RSSearch™ dataset.

The most prevalent extracranial treatment site in RSSearch™ was lung/bronchus. This correlates with the AHRQ 2011 report which described the current state of SBRT as an emerging technology for the treatment of solid malignant tumors and identified lung/thorax as the most common site treated with SBRT [59]. SBRT is now considered a standard treatment option for medically inoperable patients through prospective, multi-institutional trials [60,61]. There is a wide variety of SBRT dose and fractionation schedules reported for lung lesions which range from single fractions of 19–34 Gy [62-64] to hypofractionated schedules of 50–66 Gy delivered in 3–5 fractions [13,65-67]. In RSSearch™, the median SBRT dose to lung lesions was 54 Gy delivered in 3 fractions, which is in line with other published reports. We also reported the median maximum point dose to the esophagus, heart, trachea/bronchus and spinal cord reported in RSSearch™ and demonstrated that the median values all fell below the recommendations for normal dose limits described by the AAPM Task Group and RTOG 0618 [53]. It is reassuring to note that these tolerance doses to organs at risk were more that met without compromising on outcomes. The outcomes reported in Figures 2 and 3 from this study are comparable for this population to other studies. Moreover, these results may be more generalizable than single institution or prospective controlled studies. The results also suggest that the majority of centers participating in RSSearch™ are following the standardized treatment guidelines, published reports and protocols for SBRT for the treatment of lung cancer. Future studies are planned to obtain the entire treatment plan to correlate clinical outcomes with DVHs as well as analyze and report on the treatment management practices and clinical outcomes of lung cancer patients in RSSearch™.

The second most common extracranial site in RSSearch™ was the prostate. SBRT treatment of early stage organ-confined prostate cancer has become increasingly popular, as initial studies have demonstrated excellent biochemical control rates with very minimal toxicity [16,19,68]. In April 2013, ASTRO published their SBRT Model Policy stating that SBRT was an appropriate treatment option for select patients with low to intermediate risk prostate cancer and should be included in coverage (payment) policies [69]. Several studies have shown 5-year biochemical disease-free survival rates of 90-97% when using doses of 35 – 36.25 Gy when delivered in 5 fractions for low and intermediate risk disease. The median dose in RSSearch™ was 36.25 Gy in 5 fractions. The median max point dose to the urethra, rectum, penile bulb and femoral heads was 43, 38, 27 and 14 Gy, respectively, and fell below the published recommended normal dose tolerances (max dose of 35 – 47 Gy for urethra, 21 – 38 Gy for rectum, 50 Gy for penile bulb, and 30 Gy for femoral heads) [53,54]. The median max point dose for the bladder was 41 Gy and slightly higher than published recommendations ranging from 24 – 25 Gy. Future studies from RSSearch™ will be conducted to compare dose volume histograms of target and organs at risk to correlate clinical outcomes and toxicities.

SBRT has been evaluated for the treatment of locally advanced pancreatic with mixed results [20-23]. Variations in SBRT dose and fractionation schedules in conjunction with different chemotherapeutic agents and schedules have made the interpretation of clinical results and optimization of patient selection challenging. SBRT doses have ranged from 15 – 25 Gy in a single fraction to 24 – 45 Gy in 3 fractions with concurrent gemcitibine [20,21,23]. The University of Texas Southwest Medical Center, Dallas, TX have initiated a Phase I trial where patients with surgically resectable pancreatic cancer receive 30 Gy SBRT in 1 fraction to the surgically inaccessible at-risk margin prior to surgery, in effort to reduce local failure following surgery [70]. These studies indicate that SBRT can be an effective treatment option for pancreatic cancer, but its optimal use remains to be determined. The median SBRT dose for pancreatic lesions reported RSSearch™ was 29 Gy delivered in 3 fractions. Future studies will be conducted to investigate variations in treatment management practices and clinical outcomes for pancreatic cancer patients enrolled in RSSearch™.

The RSSearch™ registry was feasible as it was nurtured by a SRS/SBRT specialty society. Medical specialty societies are organizations that represent networks of physicians. These organizations often exist to provide services to their members in the areas of advocacy, education, and practice management. Based on their close interaction with their members and their national and international reach, medical specialty societies are uniquely positioned to collect and manage data related to the type of care their members provide. Medical specialty societies are able to connect with the members directly at meetings and symposia, and by using direct-to-member correspondences. The Radiosurgery Society, an independent non-profit organization of professionals dedicated to SRS/SBRT will foster and champion the maintenance and future success of the registry.

## Conclusion

This is the first report to describe a multi-center observational patient registry dedicated to SRS/SBRT treatments under the auspices of a medical specialty society. The RSSearch™ Registry hosts a comprehensive cache of information regarding SRS and SBRT treatments. It provides valuable information on practice patterns, procedure and patient related outcomes and serves as a valuable tool for surveillance and audit of emerging treatment modalities like SRS and SBRT. This repository would serve in comparative effectiveness research and to generate hypothesis for future research. The information in the registry may complement randomized clinical trials in a real-life clinical setting providing generalizable data. They may be useful to identify new risk factors and/or tumor characteristics that may benefit from SRS/SBRT. Future studies focusing on health-related outcomes from patients in RSSearch™ have been initiated and will be reported in subsequent analysis.

## Competing interests

Joanne Davis was previously employed by Accuray Inc. in the Department of Medical Affairs from March 2006 – January 2013. All other co-authors have no competing interests.

## Authors’ contributions

CM, SS, AS and AD contributed to patient treatment and care and data collection. JD and AM conducted the statistical analysis and drafted the manuscript. CM, SS, AS and AD participated in manuscript revisions. All authors read and approved the final manuscript.
